# Supplementary material for: Content-rich biological network constructed by mining PubMed abstracts
Source: BMC Bioinformatics. 2004 Oct 8;5:147. doi: 10.1186/1471-2105-5-147 (PMC528731; doi:10.1186/1471-2105-5-147)
Supplement: Additional File 2 — The original results of the above study (non-essential files are deleted to keep the file size under the limit set by BMC bioinformatics). [file 1471-2105-5-147-S2.bz2 › chilibotAdditionalFile2/dip50/336ID10768840E3972/html/TNFSF8_TNFRSF8.html]

 


 **TNFSF8** and **TNFRSF8** 
  
Found 158 abstracts in PubMed, retrieved 50.  
 

 What does Google say? 
 PDF only 
| .edu only 

---

**Interactive relationship** (e.g. stimulation, inhibition, etc)

**Inhibitory relationship**- Soluble CD30  [ **TNFRSF8** ]  binds to CD153  [ **TNFSF8** ]  with high affinity and blocks transmembrane signaling by CD30  [ **TNFRSF8** ] .  Ref: 11754357 Eur J Immunol, 2002
- Using a soluble CD30L  [ **TNFSF8** ]  CD8alpha chimeric protein, we assessed the ability of anti CD30  [ **TNFRSF8** ]  MAb to inhibit the binding of CD30L  [ **TNFSF8** ]  to CD30  [ **TNFRSF8** ]  expressed by the CD30  [ **TNFRSF8** ]  Karpas 299 cell line.  Ref: 10768840 Hybridoma, 2000
- Finally, we demonstrate that the anti CD30L  [ **TNFSF8** ]  MAb M81 also completely inhibits CD30  [ **TNFRSF8** ]  CD30L  [ **TNFSF8** ]  interaction.  Ref: 10768840 Hybridoma, 2000
- Proliferation of CD30  [ **TNFRSF8** ]  T Helper 2 Lymphoma Cells Can Be Inhibited by CD30  [ **TNFRSF8** ]  Receptor Cross Linking with Recombinant CD30  [ **TNFRSF8** ]  Ligand  [ **TNFSF8** ] .  Ref: 12855655 Clin Cancer Res, 2003
- The pattern of CD30L  [ **TNFSF8** ]  binding inhibition shows only limited correspondence to the functional capacity of some anti CD30  [ **TNFRSF8** ]  MAb to trigger CD30  [ **TNFRSF8** ]  signaling.  Ref: 10768840 Hybridoma, 2000
- These leukemia induced CD30  [ **TNFRSF8** ]  T cells inhibit CD40 ligand CD40L mediated S mu >S gamma and S mu >S alpha class switch DNA recombination CSR by engaging CD30  [ **TNFRSF8** ]  ligand  [ **TNFSF8** ]  CD30L  [ **TNFSF8** ] , a molecule that interferes with the assembly of the CD40 tumor necrosis factor  [ **TNFSF8** ]  receptor associated factor TRAF complex in nonmalignant IgD B cells.  Ref: 11175813 Nat Immunol, 2001
- In contrast to other members of the TNF receptor superfamily, soluble monomeric CD30  [ **TNFRSF8** ]  bound to its ligand with high affinity K D =4.5 nM and prevented the interaction of cellular CD153  [ **TNFSF8** ]  with immobilized CD30  [ **TNFRSF8** ] .  Ref: 11754357 Eur J Immunol, 2002
**Neutral relationship**- CD30L  [ **TNFSF8** ]  CD30  [ **TNFRSF8** ]  interaction is a pathway by which mast cells may stimulate DNA synthesis in HRS cells.  Ref: 11552987 Br J Haematol, 2001
- they activate HRS in vitro through CD30L  [ **TNFSF8** ]  CD30  [ **TNFRSF8** ]  interaction.  Ref: 12358914 Br J Haematol, 2002
- CD30L  [ **TNFSF8** ]  up regulates CD30  [ **TNFRSF8** ]  and IL 4 expression by T cells.  Ref: 11728464 FEBS Lett, 2001
- Mast cells express functional CD30  [ **TNFRSF8** ]  ligand  [ **TNFSF8** ]  and are the predominant CD30L  [ **TNFSF8** ]  positive cells in Hodgkin s disease.  Ref: 11552987 Br J Haematol, 2001
- Human angiogenin fused to human CD30  [ **TNFRSF8** ]  ligand  [ **TNFSF8** ]  Ang CD30L  [ **TNFSF8** ]  exhibits specific cytotoxicity against CD30  [ **TNFRSF8** ]  positive lymphoma.  Ref: 11751393 Cancer Res, 2001
- Characterization of the immunological properties of vCD30 has uncovered a role of CD30  [ **TNFRSF8** ]  CD30L  [ **TNFSF8** ]  interactions in the generation of inflammatory responses.  Ref: 12235215 J Exp Med, 2002
- These interactions include those of CD30L  [ **TNFSF8** ]  CD30  [ **TNFRSF8** ] , CD40L CD40, CD70 CD27, CD80 CD86 CD28, SCF CD117, IL 9 IL 9R, and IL 7 IL 7R.  Ref: 11725067 J Biomed Sci< MedlineTA>J Biomed Sci, 2002
- Cluster Group B antibodies, including M67 and Ki 1  [ **TNFRSF8** ] , do not affect CD30L  [ **TNFSF8** ]  binding to CD30  [ **TNFRSF8** ] .  Ref: 10768840 Hybridoma, 2000
- Soluble trimeric CD153  [ **TNFSF8** ]  bound to membrane anchored CD30  [ **TNFRSF8** ]  with a relatively high affinity K D =23 nM and was effective in triggering cell death and TNF alpha production in the presence of cross linking antibodies.  Ref: 11754357 Eur J Immunol, 2002
- We have generated soluble recombinant forms of CD30  [ **TNFRSF8** ]  and CD153  [ **TNFSF8** ]  in order to characterize their interaction and assess their biological activity.  Ref: 11754357 Eur J Immunol, 2002
- The pleiotropic functions of CD30  [ **TNFRSF8** ]  are initiated by interaction of CD30  [ **TNFRSF8** ]  expressing cells with other immune competent cells expressing CD30  [ **TNFRSF8** ]  L  [ **TNFSF8** ]  and providing the signals for modulation of effector cell activity.  Ref: 12473266 Cell Immunol, 2002
- Specific binding activity of Ang CD30L  [ **TNFSF8** ]  was verified by competition with anti CD30  [ **TNFRSF8** ]  monoclonal antibody Ki 4 and commercially available CD30L  [ **TNFSF8** ]  CD8 chimeric protein.  Ref: 11751393 Cancer Res, 2001
- These findings indicate that, in CLL, abnormal CD30  [ **TNFRSF8** ]  CD30L  [ **TNFSF8** ]  interaction impairs IgG and IgA production by interfering with the CD40 mediated differentiation of nonmalignant B cells.  Ref: 11175813 Nat Immunol, 2001
- Comparison of effects of ligation with CD30L  [ **TNFSF8** ]  and anti CD30  [ **TNFRSF8** ]  agonistic antibody HeFi 1 revealed higher efficacy for CD30L  [ **TNFSF8** ]  in these ALCL lines.  Ref: 12855655 Clin Cancer Res, 2003
- CD30  [ **TNFRSF8** ]  is a member of the tumor necrosis factor  [ **TNFSF8** ]  TNF receptor superfamily that is expressed on activated lymphocytes, as well as on neoplastic cells of Hodgkin disease HD and anaplastic large cell lymphoma ALCL.  Ref: 11110706 Blood, 2000
- The CD30 antigen  [ **TNFRSF8** ]  is a member of the tumor necrosis factor  [ **TNFSF8** ]  receptor TNFR family which is overexpressed on the surface of the tumor cells of Hodgkin s lymphoma, anaplastic large cell lymphoma ALCL, and embryonal carcinoma of the testis.  Ref: 11418184 Biochim Biophys Acta, 2001
- However, CD30  [ **TNFRSF8** ]  is cleaved at the cell surface by tumor necrosis factor  [ **TNFSF8** ]  alpha converting enzyme TACE.  Ref: 11857410 Int J Cancer, 2002
- Direct sequencing analysis of the CD30  [ **TNFRSF8** ]  and CD30L  [ **TNFSF8** ]  genes revealed four polymorphisms one in the CD30  [ **TNFRSF8** ]  gene 201G A from the transcription start site, and three in the CD30L  [ **TNFSF8** ]  gene CA repeat in the promoter, 276G A in the exon 3T C in the intron 3 IVS3 73T C.  Ref: 11960307 Genes Immun, 2002
- The persistent high levels of CD30  [ **TNFRSF8** ]  and CD30L  [ **TNFSF8** ]  expression in H RS cells suggest that the autocrine CD30L  [ **TNFSF8** ]  CD30  [ **TNFRSF8** ]  cytokine receptor loop, in addition to having the paracrine activity previously thought to exist, could play important roles in the proliferation of H RS cells.  Ref: 10908157 Lab Invest, 2000
- We suggest therefore that CD30L  [ **TNFSF8** ]  expressing MDC producing medullary epithelial cells attract CCR4 expressing thymocytes, thus favoring the CD30  [ **TNFRSF8** ]  CD30L  [ **TNFSF8** ]  interaction, and therefore the apoptosis, of cells that are induced to express CD30  [ **TNFRSF8** ]  by autoantigen activation.  Ref: 10861057 J Immunol, 2000
- Furthermore, a number of Th2 associated markers, including CD30  [ **TNFRSF8** ] , IL 4 and GATA 3, were expressed by residual T cells derived from CD30L  [ **TNFSF8** ]  AML but not from CD30L  [ **TNFSF8** ]  AML, in which the presence of the Th1 associated marker LAG 3 was documented in some cases.  Ref: 11918534 Br J Haematol, 2002
- Co expression of CD30  [ **TNFRSF8** ]  ligand  [ **TNFSF8** ]  and interleukin 4 IL 4 receptors by acute myeloid leukaemia blasts is associated with the expansion of IL 4 producing CD30  [ **TNFRSF8** ]  normal T cells.  Ref: 11918534 Br J Haematol, 2002
- We report that in H RS cells overexpression of CD30  [ **TNFRSF8** ]  leads to self aggregation, recruitment of TRAF2 and TRAF5, and NF kappaB activation, independent of CD30  [ **TNFRSF8** ]  ligand  [ **TNFSF8** ] .  Ref: 11971184 Oncogene, 2002
- The tumor necrosis factor  [ **TNFSF8** ]  receptor associated factor 1 TRAF1 participates in the signal transduction of various members of the tumor necrosis factor  [ **TNFSF8** ]  receptor TNFR family, including TNFR2, CD40, CD30  [ **TNFRSF8** ] , and the Epstein Barr virus EBV encoded latent membrane protein 1 LMP1.  Ref: 11567226 Hum Pathol, 2001
- These findings suggest that CD30  [ **TNFRSF8** ]  T cells modulate CSR as well as IgG, IgA, and IgE production by inducing reverse signaling through B cell CD153  [ **TNFSF8** ] .  Ref: 10878352 J Immunol, 2000
- The activation antigen CD30  [ **TNFRSF8** ]  Ki 1  [ **TNFRSF8** ]  which is expressed by the lymphocytes T and B is related to the nerve growth factor NFGR like the receptor of the tumor necrosis factor  [ **TNFSF8** ]  TNF and is expressed in the group of large cell anaplastic lymphoma ACLC and Hodgkin s lymphoma and in non Hodgkin s lymphoma and within the T cells of infectious Mononucleosis.  Ref: 12730648 Infez Med< MedlineTA>Infez Med, 2000
- Recombinant CD30L  [ **TNFSF8** ]  bearing cells up regulated the expression of surface CD30  [ **TNFRSF8** ]  and increased the production of IL 4 and soluble s CD30  [ **TNFRSF8** ]  by co cultured T cells.  Ref: 11728464 FEBS Lett, 2001
- PURPOSE Cutaneous anaplastic large cell lymphomas ALCLs are characterized by the expression of CD30  [ **TNFRSF8** ] , spontaneous regression of skin lesions, and increased concentration of CD30  [ **TNFRSF8** ]  ligand  [ **TNFSF8** ]  CD30L  [ **TNFSF8** ] .  Ref: 12855655 Clin Cancer Res, 2003

**Non-interactive relationship** (e.g. studied together, co-existance, homology, etc.)

- In addition the absence of CD30  [ **TNFRSF8** ]  L  [ **TNFSF8** ]  abolished clonal contraction after primary expansion and interfered with secondary expansion upon boosting.  Ref: 12538158 Ann N Y Acad Sci, 2002
- Because transforming growth factor beta TGF beta and CD30  [ **TNFRSF8** ]  ligand  [ **TNFSF8** ]  inhibit the growth of normal lymphocytes and can be detected in regressing lesions of LyP, we tested the effect of these cytokines on cell lines clonally derived from LyP in the progression to systemic lymphoma.  Ref: 11594583 Ann N Y Acad Sci, 2001
- In a recent study, a selective increase in CD30  [ **TNFRSF8** ]  ligand  [ **TNFSF8** ]  expression in regressing lesions of lymphomatoid papulosis and cutaneous CD30  [ **TNFRSF8** ]  anaplastic large cell lymphoma was shown, suggesting that activation of the CD30  [ **TNFRSF8** ]  signaling pathway may be responsible for tumor regression.  Ref: 11121138 J Invest Dermatol, 2000
- Autocrine growth regulation of CD30  [ **TNFRSF8** ]  ligand  [ **TNFSF8** ]  in CD30  [ **TNFRSF8** ]  expressing Reed Sternberg cells distinction between Hodgkin s disease and anaplastic large cell lymphoma.  Ref: 10908157 Lab Invest, 2000
- Because these foreign proteins are highly immunogenic in humans, we have developed a new CD30  [ **TNFRSF8** ]  ligand  [ **TNFSF8** ]  based fusion toxin Ang CD30L  [ **TNFSF8** ]  using the human RNase angiogenin.  Ref: 11751393 Cancer Res, 2001
- These findings were confirmed with AML blasts expressing surface CD30L  [ **TNFSF8** ] , where blocking anti CD30  [ **TNFRSF8** ]  antibodies completely abolished the release of sCD30 and reduced the production of IL 4.  Ref: 11728464 FEBS Lett, 2001
- In this study, we examined the effect of CD30  [ **TNFRSF8** ]  ligand  [ **TNFSF8** ]  CD30L  [ **TNFSF8** ]  on CD30  [ **TNFRSF8** ]  positive H RS and ALCL cells in long term cultures or in primary cultures.  Ref: 10908157 Lab Invest, 2000
- Pleiotropic signal transduction mediated by human CD30  [ **TNFRSF8** ]  a member of the tumor necrosis factor  [ **TNFSF8** ]  receptor TNFR family.  Ref: 12389614 Leuk Lymphoma, 2002
- PBL from MGUS patients and age matched controls produced comparable levels of IL 6 when activated with anti CD3 plus IL 2, and costimulation with a soluble form of CD30  [ **TNFRSF8** ]  ligand  [ **TNFSF8** ]  sCD30L CD8alpha augmented anti CD3 inducible IL 6 production similarly in both groups.  Ref: 11237552 Clin Immunol, 2001
- We conclude that only in a subset of both benign and malignant thyroid nodules the IL 6 IL 6R signal could be induced by the CD30L  [ **TNFSF8** ]  CD30  [ **TNFRSF8** ] .  Ref: 12553555 J Endocrinol Invest, 2002
- Cowpox virus encodes a fifth member of the tumor necrosis factor  [ **TNFSF8** ]  receptor family a soluble, secreted CD30  [ **TNFRSF8** ]  homologue.  Ref: 12034885 Proc Natl Acad Sci U S A, 2002
- The purpose of this study was to determine whether CD30  [ **TNFRSF8** ]  or CD30L  [ **TNFSF8** ]  genes serve as a novel susceptibility gene for type 1 diabetes in humans.  Ref: 11960307 Genes Immun, 2002
- Co expression of IL 6 and IL 6R in the epithelial follicular cells was observed solely in CD30L  [ **TNFSF8** ]  CD30  [ **TNFRSF8** ]  positive nodules 5 15 33% oncocytic adenomas.  Ref: 12553555 J Endocrinol Invest, 2002
- Upon exposure to IL 4, a critical Ig class switch inducing cytokine, Ag activated T cells express CD30  [ **TNFRSF8** ] , the CD153  [ **TNFSF8** ]  receptor.  Ref: 10878352 J Immunol, 2000
- All 3 non metastasized MTC were IL 6 IL 6R negative, and 1 3 was CD30L  [ **TNFSF8** ]  positive CD30  [ **TNFRSF8** ]  negative.  Ref: 12553555 J Endocrinol Invest, 2002
- Plasma levels of cytomegalovirus CMV specific immunoglobulin G IgG, soluble s CD30  [ **TNFRSF8** ] , sCD26 dipeptidyl peptidase IV DPP IV enzyme activity, and tumor necrosis factor  [ **TNFSF8** ]  receptor I TNFR I were assessed in human immunodeficiency virus HIV infected patients who experienced CMV retinitis CMVR as an immune restoration disease IRD during their first 6 months of highly active antiretroviral therapy HAART and in CMV seropositive, HIV infected patients with similar baseline CD4 T cell counts who had uneventful immune reconstitution.  Ref: 12085331 J Infect Dis, 2002
- CD30  [ **TNFRSF8** ] , as a member of the tumor necrosis factor  [ **TNFSF8** ]  TNF receptor family, is expressed on the surface of activated lymphoid cells.  Ref: 10793083 Am J Pathol, 2000
- Unlike dendritic cells, the CD4 CD3 cells have little CD80 or CD86 expression but do express high levels of the TNF ligands, OX40 ligand and CD30  [ **TNFRSF8** ]  ligand  [ **TNFSF8** ] .  Ref: 12753741 Immunity, 2003
- 14 15 FTC and 5 6 ATC were CD30L  [ **TNFSF8** ]  CD30  [ **TNFRSF8** ]  negative.  Ref: 12553555 J Endocrinol Invest, 2002
- Within malignancies, epithelial coexpression of CD30L  [ **TNFSF8** ]  and CD30  [ **TNFRSF8** ]  was observed in 7% of FTC, 33% of ATC, 67% of PTC, and 67% of MTC.  Ref: 11484890 Thyroid, 2001
- In 21 patients with lymphoproliferative disease of granular lymphocytes LDGL, we investigated the expression and the function of molecules belonging to TNF receptor and TNF ligand superfamilies CD30  [ **TNFRSF8** ]  CD30L  [ **TNFSF8** ] .  Ref: 10887130 Blood, 2000
- We hypothesize that CD30  [ **TNFRSF8** ]  CD30L  [ **TNFSF8** ]  interactions explain the unusual clinical behavior of cutaneous ALCLs.  Ref: 12855655 Clin Cancer Res, 2003
- Although spontaneous production by HSC M1 cells of some of these cytokines was demonstrated, the production of others was only detectable after stimulation with exogenous CD30  [ **TNFRSF8** ]  ligand  [ **TNFSF8** ] .  Ref: 11426532 Leuk Lymphoma, 2001
- The production of IL 4 in the absence of interferon gamma IFN gamma was also detected in CD3 CD30  [ **TNFRSF8** ]  T cells from CD30L  [ **TNFSF8** ]  AML.  Ref: 11918534 Br J Haematol, 2002
- Characterization of the CD30L  [ **TNFSF8** ]  binding domain on the human CD30  [ **TNFRSF8** ]  molecule using anti CD30  [ **TNFRSF8** ]  antibodies.  Ref: 10768840 Hybridoma, 2000
- IL 6 staining associated to absent expression of CD30L  [ **TNFSF8** ]  and CD30  [ **TNFRSF8** ]  was observed in 7 follicular adenomas all belonging to variants different from toxic and hyalinizing trabecular, 2 oncocytic adenomas, 5 of the 30 colloid nodules and 2 normal thyroids.  Ref: 12553555 J Endocrinol Invest, 2002
- CD153  [ **TNFSF8** ]  CD30  [ **TNFRSF8** ]  ligand  [ **TNFSF8** ]  has been described as a 40 kDa type II transmembrane glycoprotein belonging to the TNF superfamily and is expressed primarily by activated T cells, B cells and monocytes.  Ref: 12392508 Tissue Antigens, 2002
- In these cells, CD153  [ **TNFSF8** ]  engagement by an agonistic anti CD153  [ **TNFSF8** ]  mAb or T cell CD30  [ **TNFRSF8** ]  inhibits S mu >Sgamma, Smu >Salpha, and S mu >Sepsilon class switch DNA recombination CSR.  Ref: 10878352 J Immunol, 2000
- In a cell kinetic analysis we found that ALCL cell growth is effectively inhibited by CD30L  [ **TNFSF8** ]  but only in those cell lines expressing CD30  [ **TNFRSF8** ]  molecules in sufficient amounts on the cell surface.  Ref: 12855655 Clin Cancer Res, 2003
- NT and CN expressed neither CD30L  [ **TNFSF8** ]  nor CD30  [ **TNFRSF8** ]  CD30L  [ **TNFSF8** ]  CD30  [ **TNFRSF8** ] .  Ref: 11484890 Thyroid, 2001
- The results suggest that progression of LyP to lymphoma is associated with escape of lymphoma cells from growth regulation by TGF beta and CD30  [ **TNFRSF8** ]  ligand  [ **TNFSF8** ] .  Ref: 11594583 Ann N Y Acad Sci, 2001
- We suggest that sCD30 in cord blood and CD30  [ **TNFRSF8** ]  expression by decidual cells may reflect the Th2 environment surrounding the fetus, and both CD30  [ **TNFRSF8** ]  and CD30L  [ **TNFSF8** ]  could have immune regulatory functions in placenta.  Ref: 11286574 Placenta, 2001
- Presence of CD30  [ **TNFRSF8** ]  and CD30L  [ **TNFSF8** ]  cells in human placenta and soluble CD30  [ **TNFRSF8** ]  levels in cord blood are independent of maternal atopy.  Ref: 11286574 Placenta, 2001
- Tumour samples from 42 patients were immunohistochemically double stained for tryptase, a mast cell specific proteinase and CD30  [ **TNFRSF8** ]  ligand  [ **TNFSF8** ]  CD30L  [ **TNFSF8** ] .  Ref: 11552987 Br J Haematol, 2001
- Soluble CD30  [ **TNFRSF8** ]  may bind to its ligand, CD153  [ **TNFSF8** ] , with high affinity and block its interaction with membrane bound CD30  [ **TNFRSF8** ] .  Ref: 11754357 Eur J Immunol, 2002
- CD30L  [ **TNFSF8** ]  binding by CD30  [ **TNFRSF8** ]  is blocked by MAb that recognize epitopes belonging to cluster Group A like Ber H2, Ber H8, and HRS 4 as well as cluster Group C like HeFi 1 and M44.  Ref: 10768840 Hybridoma, 2000
- The authors have reported previously that the B lymphocytes of HD express CD30  [ **TNFRSF8** ]  ligand  [ **TNFSF8** ]  CD30L  [ **TNFSF8** ] , CD153  [ **TNFSF8** ] .  Ref: 11815953 Cancer, 2002
- CD30  [ **TNFRSF8** ]  CD30  [ **TNFRSF8** ]  ligand  [ **TNFSF8** ]  CD30L  [ **TNFSF8** ]  signal transduction appears to protect against autoimmune diabetes by preventing expansion of autoreactive T cells and suppressing Th1 cytokine response.  Ref: 11960307 Genes Immun, 2002
- The observation that dysregulated IgG, IgA, and or IgE production is often associated with up regulation of T cell CD30  [ **TNFRSF8** ]  prompted us to test the hypothesis that engagement of B cell CD153  [ **TNFSF8** ]  by T cell CD30  [ **TNFRSF8** ]  modulates Ig class switching.  Ref: 10878352 J Immunol, 2000
- The addition of anti CD30  [ **TNFRSF8** ] , CD30L  [ **TNFSF8** ] , CD40, CD40L, CD95, and CD95L mAbs did not show any significant effect on cell proliferation at resting conditions or after rIL 2 stimulation.  Ref: 10887130 Blood, 2000
- Of the 6 tumors arising from the parafollicular C cells medullary thyroid cancer, MTC, all 3 that had metastasized were CD30L  [ **TNFSF8** ]  CD30  [ **TNFRSF8** ]  IL 6 positive and IL 6R negative.  Ref: 12553555 J Endocrinol Invest, 2002
- Most H RS cells were CD30  [ **TNFRSF8** ]  positive, indicating co expression of CD30  [ **TNFRSF8** ]  and CD30L  [ **TNFSF8** ]  in the majority of lymphoma cells.  Ref: 10908157 Lab Invest, 2000
- Association study between CD30  [ **TNFRSF8** ]  and CD30  [ **TNFRSF8** ]  ligand  [ **TNFSF8** ]  genes and type 1 diabetes in the Japanese population.  Ref: 11960307 Genes Immun, 2002
- Co expression of interleukin 6 IL 6 and interleukin 6 receptor IL 6R in thyroid nodules is associated with co expression of CD30  [ **TNFRSF8** ]  ligand  [ **TNFSF8** ]  CD30  [ **TNFRSF8** ]  receptor.  Ref: 12553555 J Endocrinol Invest, 2002
- CD30  [ **TNFRSF8** ] , a member of the tumor necrosis factor  [ **TNFSF8** ]  receptor TNFR family, is a characteristic cell surface receptor for activated T cells and the malignant cells of Hodgkin s disease HD, anaplastic large cell lymphoma ALCL and a few other non Hodgkin s lymphomas.  Ref: 12389614 Leuk Lymphoma, 2002
- The aim of this study was to compare the presence of CD30  [ **TNFRSF8** ] , a molecule associated with Th2 related disorders such as atopic allergy, and its ligand CD30L  [ **TNFSF8** ]  in placenta in order to investigate if the placenta environment differs between atopic and non atopic women.  Ref: 11286574 Placenta, 2001
- To investigate the roles of the co stimulatory molecules CD30  [ **TNFRSF8** ]  CD30L  [ **TNFSF8** ] , CD27 CD27L, OX40 OX40L, and 4 1BB 4 1BBL, which belong to the tumor necrosis factor  [ **TNFSF8** ]  TNF receptor ligand superfamily, in the development of acute viral myocarditis, the expression of these molecules was first analysed in the hearts of mice with acute myocarditis induced by Coxsackievirus B3 CVB3 in vivo.  Ref: 11745696 J Pathol, 2001
- CD153  [ **TNFSF8** ]  CD30  [ **TNFRSF8** ]  ligand  [ **TNFSF8** ]  is a member of the TNF ligand cytokine family expressed on the surface of human B cells.  Ref: 10878352 J Immunol, 2000
- Functional expression of receptor activator of nuclear factor kappaB in Hodgkin disease cell lines.The malignant Hodgkin and Reed Sternberg cells of Hodgkin disease express several members of the tumor necrosis factor  [ **TNFSF8** ]  receptor family, including CD30  [ **TNFRSF8** ]  and CD40, and secrete several cytokines and chemokines.  Ref: 11675352 Blood, 2001
- Based on our recent data of CD30  [ **TNFRSF8** ]  ligand  [ **TNFSF8** ]  CD30L  [ **TNFSF8** ]  CD30  [ **TNFRSF8** ]  receptor CD30  [ **TNFRSF8** ]  in these nodules and on the knowledge that this signal stimulates IL 6 production in non thyroid neoplasms, we wanted to evaluate the immunocytochemical expression of these 2 ligand receptor systems in a large archival series of paraffin embedded specimens.  Ref: 12553555 J Endocrinol Invest, 2002
- The tumor necrosis factor  [ **TNFSF8** ]  receptor family molecule CD30  [ **TNFRSF8** ]  is expressed by activated and memory T cells, depending on IL 4 stimulation preferentially in association with Th0 and Th2 type responses.  Ref: 11756152 Blood, 2002
- Progression of lymphomatoid papulosis to systemic lymphoma is associated with escape from growth inhibition by transforming growth factor beta and CD30  [ **TNFRSF8** ]  ligand  [ **TNFSF8** ] .  Ref: 11594583 Ann N Y Acad Sci, 2001
- Forty percent of OA and 20% of FA showed epithelial coexpression of CD30L  [ **TNFSF8** ]  and CD30  [ **TNFRSF8** ] , and interstitial expression of CD30L  [ **TNFSF8** ] , which was also observed in the surrounding normal tissue.  Ref: 11484890 Thyroid, 2001
- In the normal thyroid tissue, colloid nodules, and another subset of benign and malignant thyroid nodules, IL 6 expression is under control of signals other than CD30L  [ **TNFSF8** ]  CD30  [ **TNFRSF8** ] .  Ref: 12553555 J Endocrinol Invest, 2002
- In conclusion, CD30L  [ **TNFSF8** ]  CD30  [ **TNFRSF8** ]  signaling is activated only past the colloid nodule stage, most frequently in an autocrine fashion.  Ref: 11484890 Thyroid, 2001
- The notion that IL 4 was able to enhance in vitro proliferation of CD30L  [ **TNFSF8** ]  IL 4R purified leukaemic blasts suggests that the selective interaction of IL 4 producing CD30  [ **TNFRSF8** ]  T cells with CD30L  [ **TNFSF8** ]  leukaemic progenitors may have a role in the progression of this particular AML subset.  Ref: 11918534 Br J Haematol, 2002
- Expression of CD30  [ **TNFRSF8** ]  ligand  [ **TNFSF8** ]  and CD30  [ **TNFRSF8** ]  receptor in normal thyroid and benign and malignant thyroid nodules.  Ref: 11484890 Thyroid, 2001
- PTC and MTC had the highest proportion of CD30L  [ **TNFSF8** ]  or CD30  [ **TNFRSF8** ]  cells, and together with OA, a thus far unreported nuclear location of CD30L  [ **TNFSF8** ] .  Ref: 11484890 Thyroid, 2001
- CD30  [ **TNFRSF8** ]  was expressed on the decidual stromal cells alone, while CD30L  [ **TNFSF8** ] , previously not described in placenta, was detected on macrophage like HLA DR cells throughout the mesenchymal chorionic villi.  Ref: 11286574 Placenta, 2001
- Characterization of the murine CD30  [ **TNFRSF8** ]  ligand  [ **TNFSF8** ]  CD153  [ **TNFSF8** ]  gene gene structure and expression.  Ref: 12392508 Tissue Antigens, 2002
- CD30  [ **TNFRSF8** ]  ligand  [ **TNFSF8** ]  CD30L  [ **TNFSF8** ] , but not its cognate receptor CD30  [ **TNFRSF8** ] , is frequently expressed on acute myeloid leukaemia AML blasts.  Ref: 11918534 Br J Haematol, 2002
- The affinity and kinetics of the interaction between soluble CD30  [ **TNFRSF8** ]  and CD153  [ **TNFSF8** ]  were determined using the BIAcore biosensor.  Ref: 11754357 Eur J Immunol, 2002
- The immunoregulatory roles of interleukin 2 IL 2, IL 4, IL 10, gamma interferon IFN gamma, tumor necrosis factor  [ **TNFSF8** ]  alpha TNF alpha, the soluble form of the IL 2 receptor sIL 2R, and the soluble form of CD30  [ **TNFRSF8** ]  sCD30 were evaluated in patients with hepatitis B virus HBV infection.  Ref: 12414777 Clin Diagn Lab Immunol, 2002
- Creating CD30  [ **TNFRSF8** ]  L  [ **TNFSF8** ]  deficient mice and studying CD8 CTL activation and memory responses, it was observed that the absence of CD30  [ **TNFRSF8** ]  L  [ **TNFSF8** ]  resulted in diminished primary clonal expansion of CD8 cells.  Ref: 12538158 Ann N Y Acad Sci, 2002
- CD30  [ **TNFRSF8** ]  and its counter receptor CD30  [ **TNFRSF8** ]  ligand  [ **TNFSF8** ]  CD30L  [ **TNFSF8** ]  are members of the TNF receptor TNFalpha superfamily and function to regulate lymphocyte survival and differentiation.  Ref: 10768840 Hybridoma, 2000
- We show that vCD30 is a 12 kD secreted protein that not only binds CD30L  [ **TNFSF8** ]  with high affinity and prevents its interaction with CD30  [ **TNFRSF8** ] , but it also induces reverse signaling in cells expressing CD30L  [ **TNFSF8** ] . vCD30 blocked the generation of interferon gamma producing cells in vitro and was a potent inhibitor of T helper cell Th 1 but not Th2 mediated inflammation in vivo.  Ref: 12235215 J Exp Med, 2002
- Engagement of CD153  [ **TNFSF8** ]  CD30  [ **TNFRSF8** ]  ligand  [ **TNFSF8** ]  by CD30  [ **TNFRSF8** ]  T cells inhibits class switch DNA recombination and antibody production in human IgD IgM B cells.  Ref: 10878352 J Immunol, 2000
- In conclusion, we could not find significant association between CD30  [ **TNFRSF8** ]  or CD30L  [ **TNFSF8** ]  genes and type 1 diabetes, but CA 9 allele in the promotor or T allele of 73T C in intron 3 in CD30L  [ **TNFSF8** ]  gene might play a minor role in the pathogenesis of type 1 diabetes, only in the Japanese female population.  Ref: 11960307 Genes Immun, 2002
- In patients and healthy subjects as control group, we determined serum levels of interleukin IL 2, interferon IFN gamma, IL 4, IL 6, IL 7, IL 8, tumor necrosis factor  [ **TNFSF8** ]  TNF alpha cytokines and soluble IL 2 receptor sIL 2R, CD30  [ **TNFRSF8** ]  sCD30, ICAM 1 sICAM 1 molecules, phenotype of peripheral blood mononuclear cells PBMC.  Ref: 10851496 Cancer Biother Radiopharm, 1996
- We screened CD30  [ **TNFRSF8** ]  and CD30L  [ **TNFSF8** ]  genes for polymorphisms in Japanese patients with type 1 diabetes and control subjects.  Ref: 11960307 Genes Immun, 2002
- A vCD30Fc fusion protein binds CD153  [ **TNFSF8** ]  CD30  [ **TNFRSF8** ]  ligand  [ **TNFSF8** ]  specifically, and it completely inhibits CD153  [ **TNFSF8** ]  CD30  [ **TNFRSF8** ]  interactions.  Ref: 12034885 Proc Natl Acad Sci U S A, 2002
- Association studies revealed no association between the CD30  [ **TNFRSF8** ]  and CD30L  [ **TNFSF8** ]  genes and type 1 diabetes in the whole population.  Ref: 11960307 Genes Immun, 2002
- Because the CD30  [ **TNFRSF8** ]  ligand  [ **TNFSF8** ]  CD30L  [ **TNFSF8** ]  CD30  [ **TNFRSF8** ]  receptor CD30  [ **TNFRSF8** ]  system is expressed in certain malignancies, but has not been studied in thyroid nodules, we investigated its immunohistochemical expression in 6 normal thyroids NT and 131 thyroid nodules 28 colloid nodules CN, 45 adenomas 15 oncocytic OA, 30 follicular FA and 58 carcinomas 15 follicular FTC, 1 insular ITC, 6 anaplastic ATC, 30 papillary PTC, and 6 medullary MTC.  Ref: 11484890 Thyroid, 2001
- In addition, engagement of T cell CD30  [ **TNFRSF8** ]  by CD30L  [ **TNFSF8** ]  on neoplastic CLL B cells down regulates the CD3 induced expression of CD40L.  Ref: 11175813 Nat Immunol, 2001
- A CD30  [ **TNFRSF8** ]  ligand  [ **TNFSF8** ]  agonist antibody caused proliferation of tumor cells from one patient and had no effect on tumor cells of another.  Ref: 11594583 Ann N Y Acad Sci, 2001
- There were no differences in the immunohistochemical expression of CD30  [ **TNFRSF8** ]  and CD30L  [ **TNFSF8** ]  in placenta from atopic n=28 compared with non atopic n=37 women.  Ref: 11286574 Placenta, 2001
- This information is useful for applying these MAbs in functional studies to further investigate the CD30  [ **TNFRSF8** ]  CD30L  [ **TNFSF8** ]  system and for designing assays for soluble CD30L  [ **TNFSF8** ] .  Ref: 10768840 Hybridoma, 2000
- In another oncocytic adenoma, another 4 conventional PTC and another 7 non conventional PTC CD30L  [ **TNFSF8** ]  CD30  [ **TNFRSF8** ]  expression was associated to expression of IL 6 only.  Ref: 12553555 J Endocrinol Invest, 2002
